# 5 Water Spray Attenuates Increases in Core Temperature During Physical Activity in Well-healed Burn Survivors

**DOI:** 10.1093/jbcr/irae036.005

**Published:** 2024-04-17

**Authors:** Craig G Crandall, Tina L Palmieri, Whitley Atkins, Josh Foster, Zachary McKenna, Luke Belval, Bonnie Orth, Joseph Watso

**Affiliations:** University of Texas Southwestern Medical Center, Dallas, Texas; UC Davis, Shriners Children's Northern California, Sacramento, CA; King's College London, London, England; University of Texas Southwestern Medical Center, Dallas, Texas; Naval Submarine Medical Research Lab, Groton, ConnecticuT; Florida State University, Tallahassee, Florida; University of Texas Southwestern Medical Center, Dallas, Texas; UC Davis, Shriners Children's Northern California, Sacramento, CA; King's College London, London, England; University of Texas Southwestern Medical Center, Dallas, Texas; Naval Submarine Medical Research Lab, Groton, ConnecticuT; Florida State University, Tallahassee, Florida; University of Texas Southwestern Medical Center, Dallas, Texas; UC Davis, Shriners Children's Northern California, Sacramento, CA; King's College London, London, England; University of Texas Southwestern Medical Center, Dallas, Texas; Naval Submarine Medical Research Lab, Groton, ConnecticuT; Florida State University, Tallahassee, Florida; University of Texas Southwestern Medical Center, Dallas, Texas; UC Davis, Shriners Children's Northern California, Sacramento, CA; King's College London, London, England; University of Texas Southwestern Medical Center, Dallas, Texas; Naval Submarine Medical Research Lab, Groton, ConnecticuT; Florida State University, Tallahassee, Florida; University of Texas Southwestern Medical Center, Dallas, Texas; UC Davis, Shriners Children's Northern California, Sacramento, CA; King's College London, London, England; University of Texas Southwestern Medical Center, Dallas, Texas; Naval Submarine Medical Research Lab, Groton, ConnecticuT; Florida State University, Tallahassee, Florida; University of Texas Southwestern Medical Center, Dallas, Texas; UC Davis, Shriners Children's Northern California, Sacramento, CA; King's College London, London, England; University of Texas Southwestern Medical Center, Dallas, Texas; Naval Submarine Medical Research Lab, Groton, ConnecticuT; Florida State University, Tallahassee, Florida; University of Texas Southwestern Medical Center, Dallas, Texas; UC Davis, Shriners Children's Northern California, Sacramento, CA; King's College London, London, England; University of Texas Southwestern Medical Center, Dallas, Texas; Naval Submarine Medical Research Lab, Groton, ConnecticuT; Florida State University, Tallahassee, Florida; University of Texas Southwestern Medical Center, Dallas, Texas; UC Davis, Shriners Children's Northern California, Sacramento, CA; King's College London, London, England; University of Texas Southwestern Medical Center, Dallas, Texas; Naval Submarine Medical Research Lab, Groton, ConnecticuT; Florida State University, Tallahassee, Florida

## Abstract

**Introduction:**

Burn injuries that require grafting impair thermoregulation, and the accompanying heightened increases in skin and core temperatures may dissuade burn injury survivors from engaging in physical activity that is beneficial for their full rehabilitation as well as cardio-metabolic health. Low-energy (i.e., non-air conditioning) cooling modalities may attenuate increases in core temperature in burn survivors, but this question has not been examined. Thus, the purpose of this study is to test the hypothesis that low-energy cooling modalities will attenuate increases in core temperature during physical activity in the heat in well-healed burn survivors.

**Methods:**

Adults with no burn injuries (non-burned; n=10), 20-40% body surface area severely burned (n=11), and >40% body surface area severely burned (n=10) walked on a treadmill for 1 hour at a low intensity (2.5 ± 0.2 mph and 2% grade) in a warm environmental chamber (39°C, 40% relative humidity). Burn survivors were at least 2 years post-injury. Testing was performed on four occasions, with the following cooling modalities applied throughout each trial (random trial order): no cooling, fan at 4 m/s (FAN), 10-50 g of water sprayed every 5 min (Water Spray; scaled to burn area size), or a combination of Water Spray+FAN. Rectal or gastrointestinal temperature (Tcore) was recorded throughout each trial.

**Results:**

None of the cooling modalities affected Tcore for the non-burned (p=0.61) or the 20-40% burn (p=0.45) groups. Moreover, FAN and Water Spray+FAN did not impact end-exercise Tcore for the >40% burn group (p≥0.48). However, in the >40% burn group Water Spray reduced end-exercise Tcore by 0.34°C (0.12 to 0.56°C) when compared with no cooling (p = 0.004).

**Conclusions:**

At the assessed workload and environmental condition, the efficacy of the applied cooling modalities on Tcore responses of burn survivors is dependent on both the selected modality and the size of the burn injury. Namely, there was no evidence that FAN or Water Spray+FAN attenuated Tcore responses regardless of the burn size, while Water Spray was effective at attenuating the elevation in Tcore in those with >40% burn injuries.

**Applicability of Research to Practice:**

These data demonstrate that skin wetting, via frequent water spraying, is effective in attenuating elevations in Tcore during physical activity in warm and humid conditions in individuals with large burn injuries. Thus, water spray may be useful to encourage such burn survivors to engage in physical activity that is beneficial for their full rehabilitation as well as cardio-metabolic health.

